# Potential genetic therapies based on m6A methylation for skin regeneration: Wound healing and scars/keloids

**DOI:** 10.3389/fbioe.2023.1143866

**Published:** 2023-04-13

**Authors:** Xiao Luo, Shu Zhu, Jia Li, Ning Zeng, Haiping Wang, Yiping Wu, Le Wang, Zeming Liu

**Affiliations:** ^1^ Department of Plastic and Cosmetic Surgery, Tongji Hospital, Tongji Medical College, Huazhong University of Science and Technology, Wuhan, China; ^**2**^ Department of Medical Ultrasound, Tongji Hospital, Tongji Medical College, Huazhong University of Science and Technology, Wuhan, China; ^**3**^ Department of Nephrology, Tongji Hospital, Tongji Medical College, Huazhong University of Science and Technology, Wuhan, Hubei, China

**Keywords:** m6A methylation, skin regeneration, wound healing, scars, keloids

## Abstract

Skin wound healing is a complex and multistage process, where any abnormalities at any stage can result in the accumulation of non-functional fibrotic tissue, leading to the formation of skin scars. Epigenetic modifications play a crucial role in regulating gene expression, inhibiting cell fate determination, and responding to environmental stimuli. m6A methylation is the most common post-transcriptional modification of eukaryotic mRNAs and long non-coding RNAs. However, it remains unclear how RNA methylation controls cell fate in different physiological environments. This review aims to discuss the current understanding of the regulatory pathways of RNA methylation in skin wound healing and their therapeutic implications with a focus on the specific mechanisms involved.

## 1 Introduction

As the protective barrier of the body, the skin is often exposed to potential causes of injury that stimulate wound healing (Zeng et al., 2021). The healing of a normal tissue wound after injury is a complex multistage process that involves hemostasis, inflammation, proliferation, and remodeling (Amjadian et al., 2022; Yu et al., 2022). It is important to note that abnormalities in any of these stages can result in the accumulation of non-functional fibrotic tissue, leading to the formation of skin scars (Amini-Nik et al., 2018; Hu et al., 2018).

A pathological scar is formed in the process of wound healing and is a proliferative disease of skin connective tissue. Keloids are one of the most common pathologic scars that occur in people of color. Keloids typically present as a bulge extending beyond the original wound, nodular hyperplasia, and a hard, red, benign mass with itching, pain, and discomfort (Jones et al., 2017; Xie et al., 2022). Excessive skin scarring, hypertrophy, or keloids pose significant challenges for patients and physicians, as they can result in serious health problems, such as contracture, functional and aesthetic issues, and complications such as pain, thickness, and itching. These problems have a significant negative impact on the physical, mental health, and social quality of life of patients and lead to high medical costs (Goel and Shrivastava, 2010). Therefore, treatment for reducing skin scarring is necessary to improve the recovery of patients.

The pathogenesis of keloids is complex and involves wound tension, genetic factors, immune changes, and programmed cell death, among other factors (Wolfram et al., 2009; Hsu et al., 2017a; Limandjaja et al., 2020; Zhang et al., 2020; Macarak et al., 2021). Keloid formation is closely related to tumor-related genes (Ekstein et al., 2021), and keloids are considered benign fibrogenic skin tumors that possess many cancer-like features, such as uncontrolled proliferation, a lack of spontaneous recovery, and high recurrence rates (Atiyeh et al., 2005). Increasing evidence suggests that the interaction among various promoting or inhibiting factors in the tumor could explain the aggressive clinical behavior of keloids. The most similar genotypes and phenotypes between keloids and cancers are cell energy sources, epigenetic methylation signatures, and epithelial–mesenchymal transformation behavior (Tan et al., 2019).

## 2 Treatment of skin regeneration

### 2.1 Wound healing

The research into the wound healing process has a rich history and is rapidly evolving. In addition to traditional therapies, novel treatment options have emerged, such as growth factors, skin substitutes, cytokine stimulants, cytokine inhibitors, matrix metalloproteinase inhibitors, gene and stem therapies, extracellular matrix therapies, angiogenic stimulants, and nanopreparation therapies (Patel et al., 2019; Qian et al., 2020; Matoori et al., 2021).

### 2.2 Scars or keloids

Scarring and keloid formation can be prevented and treated with a variety of strategies, including pressure, silicone gels, corticosteroids, lasers, and surgery. Pressure suits restrict blood flow to the scar area reducing oxygen supply, increasing collagenase activity, and reducing adhesion between collagen fibers. Stress therapy also regulates the secretion of fibrocytokines and growth factors. Silicone gels mainly increase the temperature and hydration of the blocked scar. Corticosteroids inhibit inflammation, increase the vasoconstriction of the scar, reduce collagen and glycosaminoglycan production, and decrease fibroblast proliferation. Skin grafts are effective, however, they are limited by the availability of skin, graft tissue rejection, infection, and the prevention of wound overtension (Marshall et al., 2018).

As a result, traditional treatments lack specificity, and there is no evidence to support their absolute efficacy. Even with the best treatments, traumatic scarring is inevitable, and current treatments can only reduce scarring (Ekstein et al., 2021). Therefore, it is necessary to establish new methods to prevent or reduce dermal fibrosis by optimizing the wound healing process. This requires a better understanding of the mechanisms, key regulators, and risk factors associated with wound healing (Chen et al., 2021). Recently, epigenetic changes such as DNA methylation, histone modification, and non-coding RNAs (e.g., microRNAs and long non-coding RNAs [lncRNAs]), have been recognized as promising approaches for scar management. lncRNAs, in particular, offer new potential for targeted therapy to improve traditional and combination therapies.

## 3 Epigenetic inheritance

Epigenetic modifications refer to changes in the gene expression that occur without altering the underlying DNA sequence. These modifications can be passed down from one generation to another and can be influenced by environmental factors such as diet, stress, and exposure to toxins. Epigenetic modifications play a crucial role in development, differentiation, and disease. Dysregulation of epigenetic modifications can lead to a range of disorders, including cancer, neurological disorders, and cardiovascular disease. Understanding epigenetic modifications can provide insight into disease mechanisms and potential targets for therapeutic intervention.

Epigenetic modifications including DNA deposition and histone modifications are well-established mechanisms that regulate gene expression to suppress cell fate determination and the response to environmental stimuli. DNA methylation involves the addition of a methyl group to a cytosine residue in DNA, which can result in the suppression of gene expression. Histone modification involves the addition or removal of chemical groups to histone proteins, which can affect the way that DNA is packaged and therefore impact gene expression. However, the role of transcriptional modifications (RNA) in gene expression regulation is only beginning to be revealed (Hwang et al., 2017; Livneh et al., 2020). mRNA is not just an intermediate molecule, but also an important regulator of gene expression. The process of post-transcriptional regulation of RNA involves a variety of mechanisms, both cis- and trans-acting, that are essential for controlling gene expression programs that determine cell function and fate. These mechanisms include alternative splicing, RNA editing, mRNA stability, and translation initiation, among others. Importantly, these mechanisms can be rapidly and dynamically regulated in response to changes in the cellular environment, allowing for precise control of gene expression in the face of changing conditions (Vu et al., 2019).

### 3.1 N6-methyladenosine (m6A)

m6A methylation, a prevalent post-transcriptional modification of eukaryotic mRNAs and lncRNAs, has garnered increasing attention in recent years (Wang et al., 2018). RNA methylation of transcripts was first discovered in the 1970s when a poly(A) sequence was found at the 3′end of mRNA (Desrosiers et al., 1974). However, the methylation site of m6A methylation remained in an early stage of characterization until the recent discovery of fat mass and obesity-associated protein (FTO) (Jia et al., 2011) and AlkB homolog 5 (ALKBH5) as erasers of m6A methylation (Zheng et al., 2013). These findings revealed that this modification could directly control gene expression. With the development of m6A-specific antibodies and several next-generation sequencing techniques, such as m6A sequencing (methylated RNA immunoprecipitation sequencing, meRIP-seq (Dominissini et al., 2012)), and single-nucleotide mapping of m6A methylation (miCLIP-seq (Linder et al., 2015)), the m6A modification of specific mRNAs can now be analyzed in the entire transcriptome.

m6A methylation is a widely distributed and dynamically regulated modification in the transcriptome, whose understanding is primarily focused on its “readers” and “writers.” m6A writers are methyltransferases that catalyze the transfer of methyl groups from S-adenosine methionine (SAM) to adenosine at N-6 a). The m6A “writer” complex mainly includes methyltransferase-like 3 (METTL3), METTL14, Wilms’ tumor 1-associated protein (WTAP), RNA-binding motif protein 15 (RBM15), Vir-like m6A methyltransferase associated (VIRMA)/KIAA1429, and zinc finger CCCH domain-containing protein 13 (ZC3H13) (Uddin et al., 2021), Among them, METTL3 and its homolog METTL14 form stable heterodimers and exhibit methyltransferase activity. Whereas WTAP was later identified as another component of the mammalian methyltransferase complex (Liu et al., 2021b; Oerum et al., 2021; Guo et al., 2022; Huang et al., 2022). RBM15 and its paramotifs, including RBM15B, KIAA1429, HAKAI, and ZC3H13, have also been reported in recent years, with different important functions such as recruitment and catalysis (Patil et al., 2016; Yue et al., 2018).

“Erasers” of m6A methylation refers to enzymes that can remove or reverse the addition of a methyl group to an adenosine base in RNA molecules. These enzymes are also known as demethylases (Edupuganti et al., 2017). There are several known erasers of m6A methylation, including FTO (Fat Mass and Obesity-associated protein) and ALKBH5 (AlkB Homolog 5) (Zheng et al., 2013). These enzymes belong to the family of alpha-ketoglutarate-dependent dioxygenases and use molecular oxygen to oxidize the methyl group of m6A, which is then removed as formaldehyde. FTO is a member of the AlkB family of non-heme iron and 2-oxoglutarate-dependent dioxygenases. It was the first identified m6A demethylase, and has been shown to be involved in regulating a variety of physiological processes, including adipogenesis, energy metabolism, and circadian rhythm. ALKBH5 is another m6A demethylase that has been shown to regulate RNA metabolism, including RNA splicing, translation, and decay. ALKBH5 is also involved in the regulation of various cellular processes, including cell differentiation and proliferation. (Wei et al., 2018). Future research could lead to the discovery of novel m6A demethylases and alternative mechanisms that mediate m6A modification removal and m6A-labeled transcriptional clearance.

**FIGURE 1:**
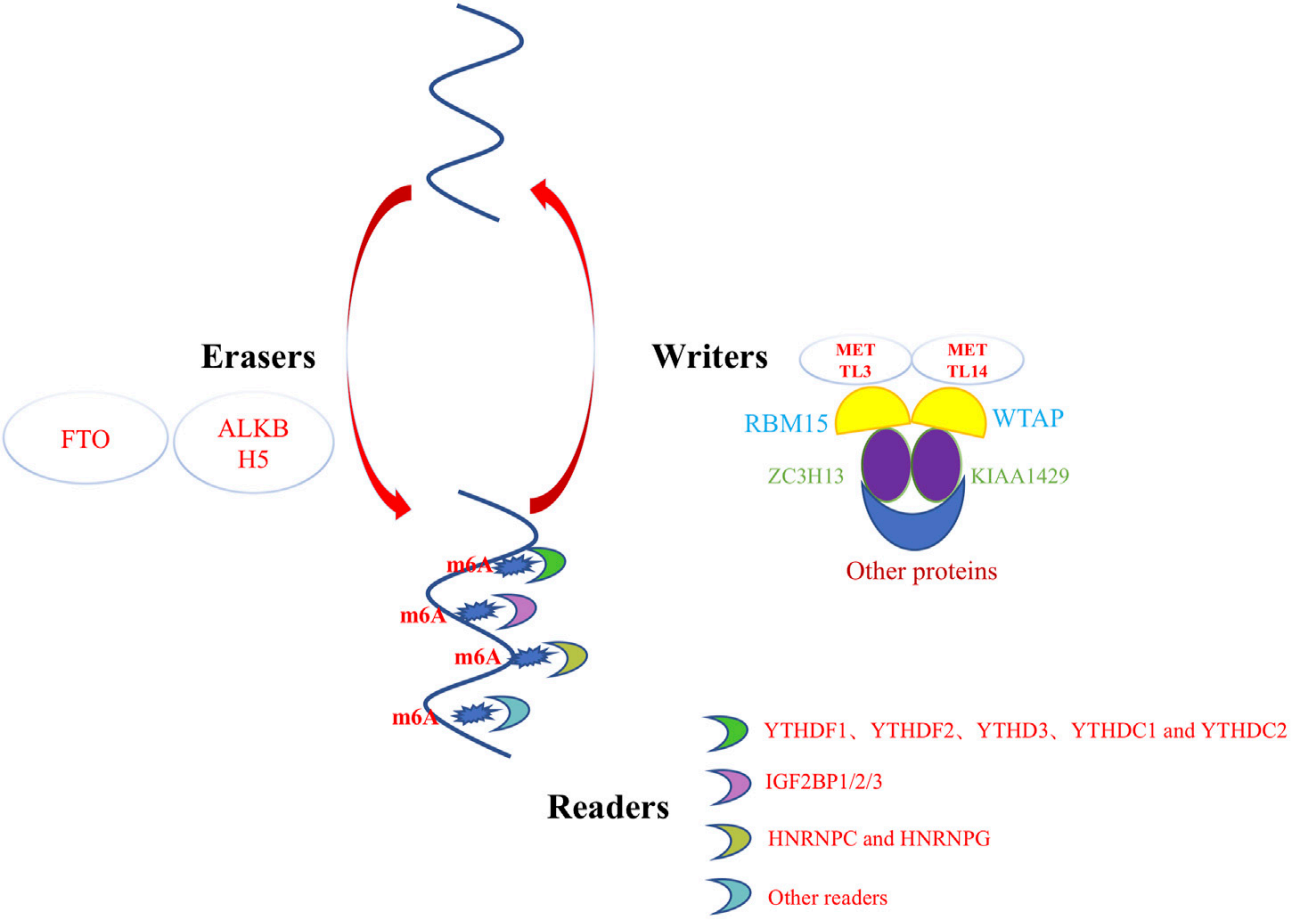
m6A methylation is dynamic regulated by writers, erasers, and readers.

The biological function of m6A modification is primarily mediated through the specific recognition and binding of readers, which are responsible for the diverse effects of m6A methylation on gene expression (Huang et al., 2020; Zhang et al., 2021b). These effects include the regulation of RNA splicing, output, decay, stabilization, and translation (Wang et al., 2014; Alarcon et al., 2015; Spitale et al., 2015; Xiao et al., 2016; Roundtree et al., 2017b; Slobodin et al., 2017). A major reader of m6A methylation is the protein family containing the YTH domain, including YTHDF1, YTHDF2, YTHD3, YTHDC1, and YTHDC2 (Luo and Tong, 2014). For instance, YTHDF1 has been found to enhance translation by binding to translation primers and ribosomes (Wang et al., 2014). In addition, the insulin-like growth factor 2 mRNA-binding protein (IGF2BP) protein family includes IGF2BP1/2/3, comprising reported readers of m6A methylation (Huang et al., 2018). Moreover, heterogeneous nuclear ribonucleoproteins C (HNRNPC) and G (HNRNPG) are considered “indirect” readers of m6A methylation because they preferentially bind to the RNA structural “switches” induced by m6A modification (Hsu et al., 2017b; Zong et al., 2021; Chen et al., 2022). For example, HNRNPC is an important physiological modulator of 3′-untranslated region processing and miRNA maturation and acts as a reader protein during m6A modification. It recognizes m6A modification groups and mediates the selective splicing of mRNA precursors (Gruber et al., 2018). Thus, RNA methylation is dynamically controlled by writers, readers, erasers, and other proteins that might affect these regulators (Figure 1). Although RNA methylation is recognized to play a crucial role in gene expression regulation and many cellular processes (Roundtree et al., 2017a; Meyer and Jaffrey, 2017; Huang et al., 2020; Livneh et al., 2020), how it controls cell fate in different physiological environments remains unclear. In skin regeneration, epigenetic modifications are essential for the proper activation and maintenance of stem cells responsible for tissue regeneration. Epigenetic changes also play a role in the differentiation of keratinocyte and fibroblast. This review aims to discuss the current understanding of the regulatory pathways of RNA methylation in skin wound healing and their therapeutic implications.

### 3.2 Role of m6A methylation in wound healing

During the wound healing process, various cellular and molecular events are activated, including the regulation of gene expression through RNA modifications such as m6A methylation. The relationship between wound healing and epigenetic inheritance is complicated and unclear (Mi et al., 2020; Zhi et al., 2021). Several studies have demonstrated that m6A methylation can affect wound healing by regulating the expression of genes involved in various cellular processes, such as proliferation, differentiation, and migration of cells (Table 1). Zhou et al. identified adipose-derived stem cells (ADSCs) via flow cytometry and tested their pluripotency in terms of differentiation into adipocytes and bone. Based on this, ADSCs were found to accelerate LEC proliferation, migration, and lymphangiogenesis through the METTL3 pathway and regulate vascular endothelial growth factor C (VEGF-C) expression and VEGF-C-mediated lymphatic angiogenesis through the METTL3/IGF2BP2-m6A pathway, thus promoting the repair of diabetic foot ulcer (DFU) wounds. The modification of ADSCs by METTL3-mediated VEGF-C m6A methylation could be a promising therapeutic strategy for promoting DFU wound healing (Zhou et al., 2021).

**TABLE 1:** Current research progresses of m6A methylation in wound healing, hypertrophic scars, and keloids.

Authors	Year	PMID	m6A protein	Type	Acting cell	Target molecule	Positive and negative effects	Type of disease
Zhi et al	2021	34753397	IGF2BP2	Reader	keratinocyte:HaCaT cell	heparanase (HPSE)	upregulation	Wound healing
Wu et al	2016	27513293	IGF2BP3	Reader	keratinocyte:HaCaT cell	let-7b	downregulation	Wound healing
Zhou et al	2021	34773968	METTL3	Writer	lymphatic endothelial cell (LEC)	VEGF-C	upregulation	Wound healing
Lee et al	2021	33729590	Mettl14	Writer	epidermal progenitor cells	Pvt1	upregulation	Wound repair
Liang et al	2021	34657574	YTHDC1	Reader	keratinocyte cells	SQSTM1	upregulation	Wound healing
Xie et al	2021	36760238	FTO	Eraser	fibroblasts	COL1A1	upregulation	Keloid
Liu et al	2021	34727293	METTL3	Writer	Tenon’s capsule fibroblasts	Smad3	upregulation	Glaucoma

Impaired physiological functions of keratinocytes induced by a high-glucose environment lead to delayed healing of diabetic wounds (Hu and Lan, 2016). In a study of diabetic skin wound healing by Liang et al., m6A RNA modification and the regulation of autophagy were found to play a key role in diabetic skin wound healing. The m6A reader protein YTHDC1 (YTH domain 1), which interacts with autophagy receptor *SQSTM1* mRNA, is downregulated in keratinocytes in both acute and chronic hyperglycemia. The knockdown of YTHDC1 affects keratinocyte biological functions, including an increased apoptosis rate and impaired wound-healing ability. Further studies found that YTHDC1 regulates autophagy in diabetic keratinocytes by regulating the stability of *SQSTM1* nuclear mRNA, ultimately affecting diabetic skin wound healing functions (Liang et al., 2022). Overall, the evidence suggests that m6A methylation plays an important role in regulating gene expression and cellular processes involved in wound healing, making it a promising target for future therapeutic interventions.

### 3.3 m6A methylation and hypertrophic scars

Hypertrophic scars (HSs) frequently arise following burns and trauma, posing a formidable challenge in the field of wound repair and plastic surgery. Conventional methods, such as surgery, radiotherapy, and hormone therapy, have proven insufficient in achieving complete HS remission, particularly in regions susceptible to HS recurrence (Jaloux et al., 2017), Current studies generally support the pathological features of HSs, including abnormal inflammation, excessive proliferation and differentiation of fibroblasts, increased angiogenesis, and excessive deposition of extracellular matrix (ECM); however, the causative factors and molecular mechanisms underlying the unremitting collagen synthesis in HSs remain indeterminate (Keane et al., 2018; Roderfeld, 2018; Zhang et al., 2021a). Whether m6A methylation, as a mechanism of targeting abnormal epigenetic modification, can serve as a new target and mechanism for fibrotic diseases and provide a new therapeutic strategy for the treatment of skin fibrosis remains to be determined.

Liu et al. (2021a) conducted a study to investigate the mechanism of m6A modification in HSs and normal skin tissues. The study employed m6A methylation and RNA sequencing, followed by bioinformatics analysis, immunoprecipitation of m6A-related RNA, real-time quantitative polymerase chain reaction verification, and other methods. The presence of 14,791 new m6A methylation peaks in HS samples was accompanied by the disappearance of 7,835 peaks. Unique m6A-related genes in HS were associated with fibrosis-related pathways, and differentially expressed mRNA transcripts were identified in HS samples with hypermethylated or hypomethylated m6A methylation peaks. The m6A transcriptome map of human HS helped to elucidate the possible mechanism underlying m6A-mediated gene expression regulation (Liu et al., 2021b).

Furthermore, Liu et al. (2021b) reported the m6A methylation mechanism of scar formation caused by human Tenon’s fibroblasts (HTFs). First, they isolated and identified primary HTFs and found that transforming growth factor beta 1 (TGF-β1) enhanced cell viability and proliferation and ECM deposition in HTFs. Subsequently, TGF-β1 was found to increase the number of m6A methylation events and promote the expression of an m6A “reader.” The study further revealed that the downregulation of METTL3 inhibited the TGF-β1-induced promotion of cell viability and proliferation and ECM deposition in HTFs. The results indicated that METTL3 can indeed regulate the expression of Smad3, playing a critical role in the regulation of TGF-β1-induced HTFs and providing new theoretical strategies for regulating scar formation based on METTL3 (Liu et al., 2021b).

### 3.4 m6A methylation and keloids

The emergence of a public database of collaborative studies has made a vast amount of gene expression data from RNA sequencing available not only for tumor tissues, but also for keloid-associated tissues. This provides great convenience for exploring and identifying gene expression differences in keloid tissue analysis. For example, tumor suppressor genes, such as p53, p16, Fas, and p27, were found to lose their inhibitory effect on fibroblast proliferation after mutations (Tan et al., 2019). Moreover, the overexpression of c-MYC and c-FOS promotes fibroblast proliferation and inhibits apoptosis (He et al., 2017).

Recently, Xie et al. (2022) collected and integrated keloid-related sequencing results from public datasets (GSE44270 and GSE145725) to establish a new keloid risk diagnosis model, extracted m6A-related genes from the literature, and compared their expression matrices between high-risk and low-risk groups to explore differences in m6A methylation. It was found that ALKBH5, FTO, and HNRNPA2B1 were highly expressed, while YTHDF2 was lowly expressed in the high-risk keloid group (Xie et al., 2022). This study provides an idea for treatment planning based on clinical risk grouping.

Furthermore, Lin et al. (2022) investigated the overall m6A-modified RNA pattern and the possible mechanism of keloid pathogenesis (Lin et al., 2022). They collected 14 pairs of normal skin and keloid tissues and found that WTAP and METTL3 protein expression was higher in keloid tissues than in normal tissues. MeRIP and RNA sequencing and bioenrichment analysis revealed 21,020 unique m6A methylation peaks and 6,573 unique m6A-related gene transcripts in keloid samples. In normal tissues, 4,028 unique m6A methylation peaks were found, including 779 m6A-related modified genes. Subsequent functional verification also showed that the m6A-methylated genes and the differentially upregulated genes between the two tissues were mainly related to the Wnt signaling pathway. These findings directly confirmed that keloid fibroblasts were in a state of m6A methylation activation and that the high expression of the Wnt/β-catenin pathway in skin fibroblasts, modified and activated via m6A methylation, might promote the occurrence of keloid.

## 4 Conclusions and perspectives

Wound healing is a complex biological process involving a series of molecular events to promote skin regeneration. However, abnormal wound healing can lead to the formation of thick, painful, and itchy scars, which can cause aesthetic and functional complications. Therefore, it is crucial to develop more effective and targeted treatments to prevent the deposition of excess fibrous tissue. This can be achieved through a correct understanding of the regulatory mechanisms of wound healing and scar formation.

Given the significant role of genetic and epigenetic differences in physiological or pathological wound repair, we emphasize the critical role of m6A epigenetic changes in regulating the switch during wound healing. Regulatory epigenetic molecules are considered promising therapeutic tools for scar management. However, their exact roles need to be further explored in terms of unexpected genetic disorders.

In conclusion, recent studies have suggested that RNA methylation is a major pathway regulating skin wound healing. This finding highlights the therapeutic potential of genetic and epigenetic approaches for optimizing wound healing and scar management. However, the precise function of m6A methylation in regulating skin wound healing at the cellular level and its potential mechanism in regulating gene expression after transcription require further investigation. Whereas it is exciting to explore RNA methylation as a promising avenue from a therapeutic perspective, translation of these new findings to the clinic remains an urgent challenge. Successful application of these emerging strategies will require advances in existing tools and technologies for the discovery, delivery, and control of genetic and epigenetic regulation, which can help overcome associated challenges and provide a new approach to wound healing management.

## Abbreviations

lncRNAs, long non-coding RNAs; ALKBH5, AlkB homolog 5; meRIP-seq, methylated RNA immunoprecipitation sequencing; miCLIP-seq; single nucleotide mapping of m6A; SAM, S-adenosine methionine; METTL3, methyltransferase-like 3; METTL14, methyltransferase-like 14; WTAP, Wilms’ tumor 1-associated protein; RBM15, RNA-binding motif protein 15; VIRMA, Vir-like m6A methyltransferase associated; ZC3H13, Zinc finger CCCH domain-containing protein 13; HNRNPC, heterogeneous nuclear ribonucleoproteins C; VEGF-C, vascular endothelial growth factor C; ECM, extracellular matrix; TGF-β1, transforming growth factor beta 1.
